# Physicians’ perceptions and treatment practices for agitation associated with Alzheimer’s dementia vary by specialty in Japan

**DOI:** 10.1038/s41598-026-51118-5

**Published:** 2026-05-07

**Authors:** Shunichiro Shinagawa, Keisuke Onuki, Koichi Shimizu

**Affiliations:** 1 Department of Psychiatry, The Jikei University School of Medicine, 3-25-8 Nishi-Shinbashi, Minato-ku, Tokyo 105-8461 Japan; 2 Department of Medical Affairs, Otsuka Pharmaceutical Co., Ltd., Shinagawa Grand Central Tower, 2-16-4 Konan, Minato-ku, Tokyo 108-8242 Japan; 3 Department of Public Health, Graduate School of Juntendo University, 2-1-1 Hongo, Bunkyo-ku, Tokyo 113-8421 Japan

**Keywords:** Aberrant Motor Behavior in Dementia, Alzheimer disease, Perception, Surveys and Questionnaires, Therapeutics, Diseases, Health care, Medical research, Neurology, Neuroscience

## Abstract

Agitation, a behavioural and psychological symptom of dementia, is under-recognized in Japan. To describe the physician’s perceptions and treatment practice for agitation in Alzheimer’s dementia (AAD) in Japan, we conducted a cross-sectional web-based survey in October 2024. The survey included physicians in neurology, neurosurgery, psychiatry, or general internal medicine who were registered with the survey panel; consented to participation; affiliated with hospitals or clinics; treating ≥ 10 people with Alzheimer’s dementia (AD)/month. Responses from 529 physicians showed that they treated an average of 35.0 people with AD per month, of whom 8.4 (24%) had AAD. When asked what “agitation” brought to mind, physicians most commonly selected the Japanese term for “excitability”, corresponding to the “agitation/aggression” item in the Neuropsychiatric Inventory (58.6%). In general internal medicine, 24.2% were unaware of agitation. Anti-dementia drugs (91.7%) were most frequently selected as new medications for AD, whereas in psychiatry, antipsychotics were most frequently selected (95.8%), and side effects were cited more often as a key consideration than in other specialties. These results suggest that perceptions and treatment practices vary by specialty, particularly reflected in common antipsychotic prescriptions with higher safety awareness among psychiatrists and limited recognition of AAD in others, especially in general internal medicine.

**Supplementary Information:**

The online version contains supplementary material available at 10.1038/s41598-026-51118-5.

## Introduction

In Japan’s super-aged society, the prevalence of people with dementia is estimated to be 12% among older adults and is projected to reach 5.23 million in 2030 and 5.87 million in 2050^1^. As Alzheimer’s dementia (AD) accounts for 60–80% of all dementia diagnoses^2^, the prevalence of AD is expected to increase correspondingly. The core feature of AD is the progressive decline of cognitive function. However, people with AD exhibit various behavioural and psychological symptoms of dementia (BPSD), including apathy, delusions, anxiety, hallucinations, agitation, misidentifications, aggression, depression, wandering, sundowning, hypersexuality, disinhibition, and catastrophic reactions^3^. BPSD are highly prevalent, reported in 99% of people with AD^4^, and significantly impacts people and caregivers.

Agitation is one of the most common BPSD^5^. In a study using a US electronic health record database, approximately 45% of people with AD had agitation over a two-year observation period^6^. The International Psychogeriatric Association (IPA)^7^ defines agitation as a behavioural symptom observed in individuals with cognitive impairment including dementia; characterized by persistent or markedly altered behaviour of excessive motor activity, verbal aggression, or physical aggression; accompanied by emotional distress resulting in disability beyond that expected from cognitive impairment; and not solely explained by other medical, psychiatric, or environmental conditions. Typical behaviours include restlessness, pacing, yelling, screaming, grabbing, and destroying objects. Agitation also places a burden on caregivers and society in terms of an increased need for caregiver presence, greater healthcare resource utilization (e.g., healthcare professional consultations, hospital or nursing home admissions, and antipsychotic use), and higher direct medical costs^8^. Increased burden further diminishes work productivity and impairs caregivers’ daily activities in a severity-dependent manner^9^. Therefore, early detection and appropriate treatment for agitation in AD (AAD) are crucial for improving prognosis and quality of life, and alleviating caregiver’s burden.

Agitation is not widely recognized in Japanese clinical practice, partly for linguistic reasons. Although the term “agitation” has been introduced into Japanese, it is not commonly used and is often replaced by the more familiar Japanese word “*Kofun*”. However, “*Kofun*” has broader meanings and is not restricted to pathological states; it can also denote general excitability or ordinary emotional reactions. Therefore, it does not necessarily correspond to the clinical concept of agitation. Additionally, although current guidelines^10^ offer overall guidance regarding BPSD treatment, prioritizing non-pharmacological management, standardized diagnostics and treatment approaches specific to AAD have not been fully established in Japan. Consequently, diagnosis and treatment of AAD often rely on physician’s discretion^11^. Until the recent approval of brexpiprazole in 2024 for “excessive motor activity or physically/verbally aggressive behaviour due to rapid changes in mood, irritability, and/or outbursts associated with dementia due to Alzheimer’s disease”^12^, pharmacological options for AAD in Japan had been only off-label uses with limited evidence supporting their efficacy and safety. The limited recognition of the term “agitation” may underlie the avoidance of explicit use of the term in the official indication. These circumstances pose challenges to ensuring consistent practices among clinicians and across specialties. Given these uncertainties and paucity of evidence regarding AAD in Japanese clinical practice, we considered it important to investigate clinicians’ perspectives on AAD and its actual treatment.

Therefore, we conducted a web-based survey to describe real-world physicians’ perceptions and treatment practices, including prescription behaviours, for AAD, focusing on the characteristics by medical specialties. Clarifying AAD clinical practice in Japan may inform optimal care strategies with medical and societal implications.

## Methods

### Study design and survey participants

This cross-sectional web-based survey was conducted from October 15 to October 18, 2024, by INTAGE Healthcare Inc. (Tokyo, Japan), using the online survey panel of physicians provided by PLAMED Inc. (Tokyo, Japan). Physicians voluntarily registered to the panel; upon registration, medical license was verified. As of August 2023, PLAMED survey panel comprised approximately 60,000 physicians across 35 specialties in Japan. Approximately 21.0% of physicians in psychiatry (650/3,100), 15.4% in neurology (200/1,300), 5.9% in neurosurgery (100/1,700), and 9.1% in general internal medicine (700/7,700) were treating more than 10 people with AD. Target sample size was planned based on response rates in previous surveys conducted by INTAGE Healthcare Inc. using the same survey panel.

Randomly selected panel registrants (*n* = 3,560) were invited via an email containing the survey link. Those who consented to the survey online (*n* = 576) proceeded to a screening, which included questions about demographic and facility characteristics (e.g., age, specialties, and number of people with AD the physicians were treating). Among those who did not proceed to the screening, four individuals did not provide consent, 64 discontinued the consent process, and the remaining 2,916 individuals did not access the survey link or dropped out before accessing the consent form.

Participants were included in the main survey if their screening responses indicated affiliation with the hospitals or clinics, treatment of ≥ 10 people with AD in the past month, and if specialty was neurology, neurosurgery, psychiatry, or general internal medicine. No exclusion criteria were applied.

### Survey

Eligible participants proceeded directly to the main survey. The main survey included questions about practice characteristics of AD (e.g., practice setting and number of people with AAD treated in the past month), perception and usage of AD and BPSD measures and definitions, perception of BPSD and AAD, and prescription behaviour for AD and AAD.

To explore perception of BPSD, we asked participants to select the frequency of BPSD observed in people with AD and indicate which symptoms were challenging for caregivers and participants. We also assessed the perception of AAD by asking participants what came to mind when they heard the term, “agitation.” For both questions, the response options were based on symptom terms in the Japanese version of the Neuropsychiatric Inventory (NPI). “Agitation/aggression” in the English version of the NPI was presented in the survey as “*Kofun*,” consistent with the translation in the Japanese version. The closest parallel of the Japanese *“Kofun”* in English is “excitability.” To avoid confusion, this article refers to the item using its original English label, “agitation/aggression (NPI).” In addition to the existing NPI items, the survey also included delirium as a response option for the question about perception of AAD. For reference, according to IPA’s definitions^13^, “disinhibition,” “irritability/lability,” “aberrant motor behaviour,” and “agitation/aggression (NPI)” among the NPI-based symptom responses correspond to “agitation.”

To explore prescription behaviour for AD, participants were asked to indicate newly prescribed medications and reasons for not prescribing antipsychotics. Regarding prescription behaviour for AAD, participants reported prescription of antipsychotics, criteria for initiation and discontinuation, duration of prescription, key considerations, and side effects concerned upon prescribing antipsychotics.

The IPA definition of AAD was available on the survey screen to aid responses, except for questions about the perception of AAD. Participants received redeemable points (equivalent to 1,500 Japanese yen) upon completing the survey.

### Statistical analysis

This was a descriptive study summarizing the survey responses, using numbers and percentages for categorical data, and means, standard deviations (SD) and/or medians for continuous data. Survey participants were excluded from the analysis if: the age reported in the screening differed from that registered in the survey panel, the response duration was extraordinarily shorter than that of other participants, they chose the same responses across the survey, or the numerical values deviated to an unrealistic degree beyond a clinically plausible range compared to other participants. Such potential cases were evaluated by the investigators, and responses that were considered sufficiently unreliable were excluded. While the number of people with AAD was summarized for the overall analysis set, most other responses were summarized by specialty. As no missing responses were allowed, no missing imputation was conducted. The analysis was conducted using Lyche-Epoch Ver.1.10.0.0, which was originally developed by INTAGE Inc.

### Ethics approval and consent to participate

This study was approved by the independent Ethics Committee of Otsuka Pharmaceutical Co., Ltd. (approval no.241001). This study was conducted in accordance with the Declaration of Helsinki and Ethical Guidelines for Medical and Biological Research Involving Human Subjects. Participants provided informed consent to survey participation electronically, and the obtained data were fully anonymized before provision to the study investigators.

## Results

### Survey participants’ characteristics

Of the 576 individuals who consented, 533 participants completed the main survey. Four participants were excluded from the analysis because they reported treating 2,000 or more patients in the past month, which was considered unreliable and beyond the clinically plausible range for a single physician. Therefore, the analysis set comprised 529 physicians.

Males accounted for 89.2% of the analysis set [Table 1]. The most prevalent age groups were the 50s (27.2%) and 40s (24.8%). Psychiatry accounted for the largest proportion of the analysis set (40.1%), followed by general internal medicine (29.7%), neurology (19.5%), and neurosurgery (10.8%). The majority worked at general hospitals (52.2%) and hospitals with 200–499-bed capacity (34.0%).

**Table 1:** Baseline characteristics.

Characteristics		Total (*N* = 529)	
		*n*	(%)
**Gender**	Male	472	(89.2)
	Female	57	(10.8)
**Age category**	20s	13	(2.5)
	30s	105	(19.8)
	40s	131	(24.8)
	50s	144	(27.2)
	60s	113	(21.4)
	≥ 70s	23	(4.3)
**Specialty areas**	Neurology	103	(19.5)
	Neurosurgery	57	(10.8)
	Psychiatry	212	(40.1)
	General internal medicine	157	(29.7)
**Number of hospital beds**	0	131	(24.8)
	1 to 19	9	(1.7)
	20 to 99	31	(5.9)
	100 to 199	82	(15.5)
	200 to 499	180	(34.0)
	≥ 500	96	(18.1)
**Facility type**	Clinic	140	(26.5)
	University hospital	55	(10.4)
	National/public hospital	58	(11.0)
	General hospital	276	(52.2)
**Stand-alone psychiatric hospital** ^**a**^	Yes	132	(71.4)
	No	53	(28.6)

Percentages may not sum up to 100% due to rounding.

^a^ A psychiatric hospital with no other specialties; this question was displayed to only physicians specializing in psychiatry with hospital capacity of 20 beds or more (*n* = 185).

### Practice characteristics of AD and BPSD

#### Practice setting

The physicians reported treating a mean (SD) of 315.4 (273.9) people per month regardless of diseases, including 35.0 (33.5) people with AD, with the median being smaller than the mean (median of overall people treated: 250; people with AD: 23). Those in neurology reported treating the largest number of people with AD (mean [SD]: 38.8 [35.1]; median: 25), followed by psychiatry (36.4 [33.1]; 26.5), neurosurgery (31.9 [38.8]; 20), and general internal medicine (31.9 [30.8]; 20). Based on the responses from the physicians, among people with AD, the mean (SD) number of those with AAD was 8.4 (11.0), with a median of five. The corresponding ratio of the mean number of patients with AAD to those with AD reported by physicians was 24% (8.4/35.0). Overall, and across all specialties, the majority of people with AD were outpatients (29.9%, 13.0%, and 9.5% of the physicians indicated that 91–100%, 71–80%, and 81–90% of people with AD, respectively, were outpatients). Among the physicians working in clinics (*n* = 140), 95.0% indicated that they had collaborative relationships with specialized dementia centres (neurology: 93.3%, neurosurgery: 88.9%, psychiatry: 100.0%, and general internal medicine: 94.4%).

#### Perception of AD and BPSD measures and definitions

**Fig. 1:**
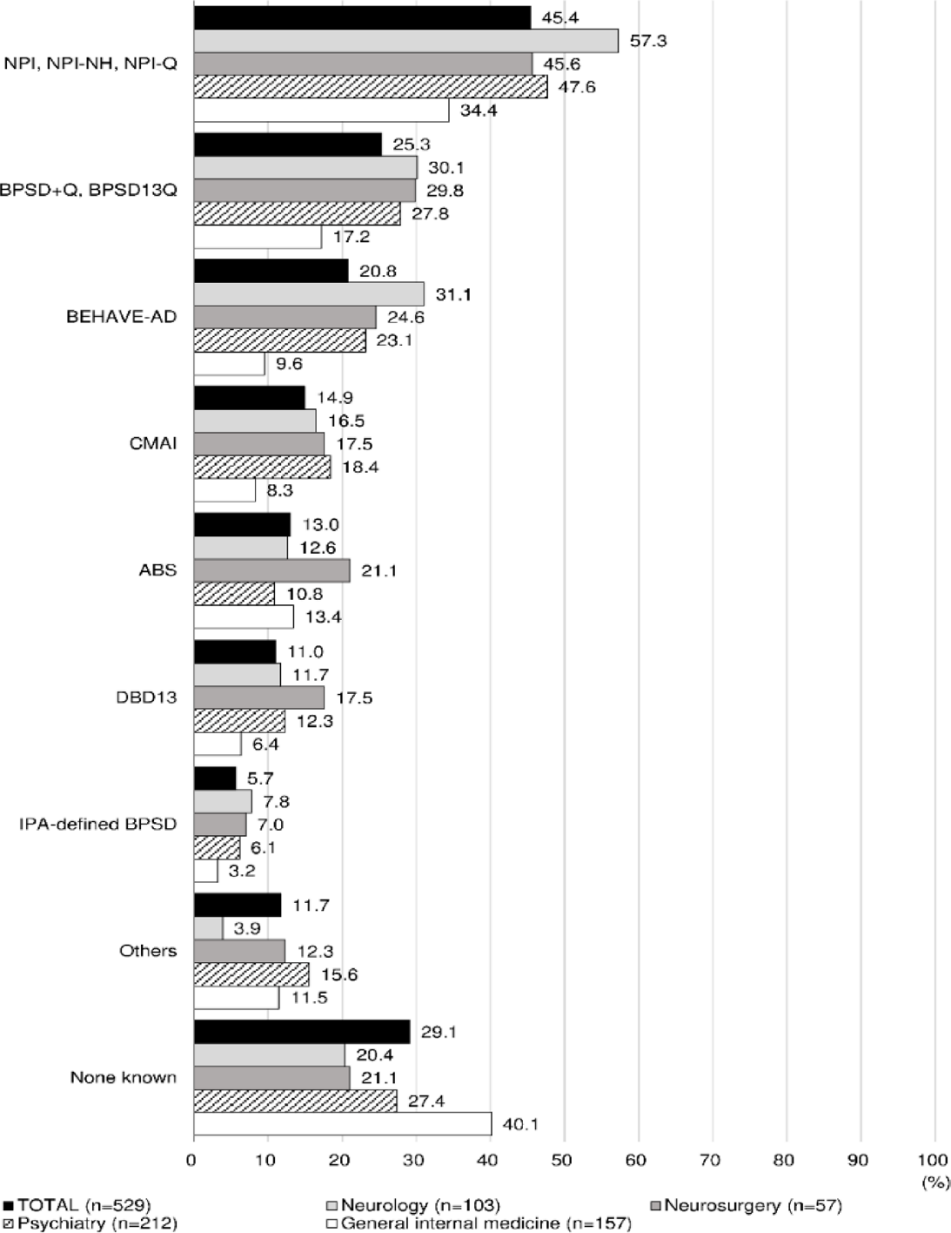
Perception of AD/BPSD measures and definitions in the analysis population (*N* = 529). Physicians were asked to select all the AD-related measures and definitions that they were aware of from the given choices. NPI, Neuropsychiatric Inventory; NPI-NH, NPI-Nursing Home Version; NPI‐Q, NPI-Questionnaire; BPSD, Behavioral and Psychological Symptoms of Dementia; BPSD + Q, BPSD Questionnaire; BPSD13Q, BPSD Questionnaire 13 item version; BEHAVE-AD, Behavioral Pathologic Rating Scale for Alzheimer’s disease; CMAI, Cohen-Mansfield Agitation Inventory; ABS, Abe’s BPSD score; DBD13, Dementia Behavior Disturbance scale; IPA, International Psychogeriatric Association.

Among the included measures and definitions, listed in Fig. 1, NPI measures (NPI, NPI-Nursing Home Version, and NPI-Questionnaire) were the most widely recognized in the analysis set (45.4%) and across specialties. Meanwhile, 29.1% of the analysis set were unaware of the measures and definitions; this percentage was particularly high in general internal medicine (40.1%).

Overall, 21.0% of the participants were aware and using the symptom measures, while 42.0% were aware but not using them, and 37.1% were not aware of them. By specialty, 24.3% of neurology, 26.3% of neurosurgery, and 21.7% of general internal medicine reported awareness and usage. The percentage was lowest in psychiatry at 17.5%; the remaining 82.5% did not use these measures (46.2% were aware but did not use them, and 36.3% were unaware).

#### Perception of BPSD

In the overall analysis set, participants reported that frequently observed BPSD included “agitation/aggression (NPI)” (71.5%), “irritability/lability” (68.4%), “nighttime behaviours” (65.4%), and “delusions” (62.2%); this was also the case across specialties [Table 2]. The physicians’ response showed that caregivers also identified similar symptoms as challenging to manage, that is, “agitation/aggression (NPI)” (59.4%), “aberrant motor behaviour” (55.2%), “nighttime behaviours” (50.5%), “irritability/lability” (44.4%), and “delusions” (39.3%). The physicians also considered similar symptoms challenging: “aberrant motor behaviour” (49.1%), followed by “agitation/aggression (NPI)” (35.4%), and “nighttime behaviours” (33.8%) [Table 2].

**Table 2:** BPSD frequently observed in AD and difficult to manage for physicians (*N* = 529).

BPSD	TOTAL (*N* = 529)	Neurology (*N* = 103)	Neurosurgery (*N* = 57)	Psychiatry (*N* = 212)	General internal medicine (*N* = 157)
**Delusions**					
Frequently observed^a^	62.2	63.1	54.4	72.6	50.3
Difficult to manage^b^	28.7	43.7	22.8	23.1	28.7
**Hallucinations**					
Frequently observed^a^	37.1	40.8	28.1	41.0	32.5
Difficult to manage^b^	22.9	29.1	26.3	14.2	29.3
**Agitation/Aggression**					
Frequently observed^a^	71.5	65.0	68.4	82.5	61.8
Difficult to manage^b^	35.4	41.7	35.1	28.3	40.8
**Depression/Dysphoria**					
Frequently observed^a^	46.3	51.5	45.6	45.3	44.6
Difficult to manage^b^	13.2	17.5	7.0	14.6	10.8
**Anxiety**					
Frequently observed^a^	57.5	52.4	59.6	62.7	52.9
Difficult to manage^b^	9.6	13.6	5.3	9.9	8.3
**Elation/Euphoria**					
Frequently observed^a^	11.9	15.5	10.5	11.3	10.8
Difficult to manage^b^	4.2	6.8	7.0	2.4	3.8
**Apathy/Indifference**					
Frequently observed^a^	57.1	60.2	56.1	57.1	55.4
Difficult to manage^b^	22.7	25.2	19.3	29.7	12.7
**Disinhibition**					
Frequently observed^a^	50.7	55.3	56.1	58.5	35.0
Difficult to manage^b^	29.1	42.7	21.1	34.4	15.9
**Irritability/Lability**					
Frequently observed^a^	68.4	65.0	68.4	83.5	50.3
Difficult to manage^b^	27.2	26.2	31.6	25.9	28.0
**Aberrant motor behaviour**					
Frequently observed^a^	58.6	50.5	42.1	65.6	60.5
Difficult to manage^b^	49.1	50.5	50.9	47.6	49.7
**Nighttime behaviours**					
Frequently observed^a^	65.4	62.1	52.6	73.1	61.8
Difficult to manage^b^	33.8	40.8	31.6	26.9	39.5
**Appetite and Eating abnormalities**					
Frequently observed^a^	28.0	26.2	17.5	30.2	29.9
Difficult to manage^b^	26.3	27.2	21.1	32.5	19.1
**No BPSD is difficult to manage** ^**b**^	1.9	0.0	1.8	0.9	4.5

The table summarizes the percentage of physicians who selected each response within each specialty.

The response options presented in the actual survey were consistent with the symptom terms derived from the Japanese version of the NPI. Particularly, the item labelled “agitation/aggression” in the English version of the NPI was presented in the survey as “*Kofun*,” consistent with the translation used in the Japanese version.

^a^ Physicians rated the frequency of each BPSD observed in their people with AD on a 5-point Likert Scale: “very low,” “low,” “neither low or high,” “high,” and “very high.” We aggregated the responses of “high” or “very high” as “Frequently observed”.

^b^ Physicians were asked to select all BPSD that they regarded challenging to manage from the symptom-related response options, as well as an option indicating “no BPSD is difficult to manage.”

BPSD, Behavioral and Psychological Symptoms of Dementia; AD, Alzheimer’s dementia.

### Perception of AAD

**Fig. 2:**
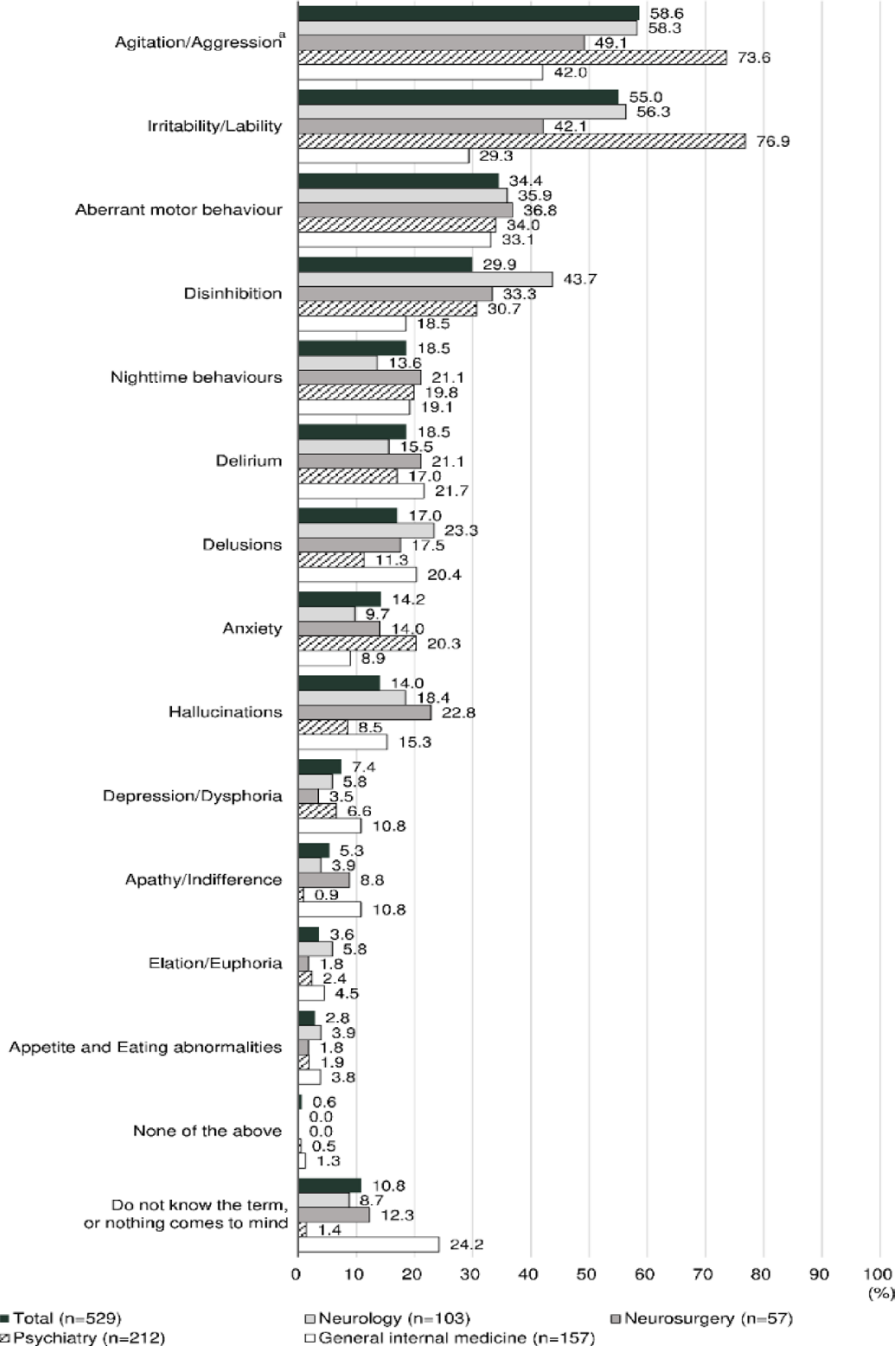
Perceived symptoms associated with “agitation” (*N* = 529). Physicians were asked to select what came to mind when they heard the term “agitation” from the symptoms and options. The symptom-related response options presented to the participants were consistent with the symptom terms derived from the Japanese version of the NPI. In addition to the original NPI items, delirium was also included in the response items. ^a^ Particularly, the item labelled “agitation/aggression” in the English version of NPI was presented in the survey as “Kofun,” consistent with the translation used in the Japanese version.

Among the symptoms listed in Fig. 2, “agitation/aggression (NPI),” which was displayed as “*Kofun*” in accordance with the Japanese NPI, was the most frequently selected (58.6%) as the symptom associated with the term, “agitation.” In psychiatry, “irritability/lability” (76.9%) was cited more than “agitation/aggression (NPI)” (73.6%), with higher percentages (more than 20%) than in other specialties. In general internal medicine, 24.2% answered that they “do not know the term or nothing comes to mind.”

### Prescription behaviour for AD

#### AD treatment

When asked to select all medications they had newly prescribed for AD, physicians selected anti-dementia drugs (91.7%) most frequently, both overall and across most specialties, followed by hypnotics (82.0%). This differed in psychiatry, where antipsychotics were most frequently selected (95.8%), followed by anti-dementia drugs (91.5%). The selection of anxiolytics was slightly less common in psychiatry (39.6%) than in other specialties (neurology: 56.3%, neurosurgery: 42.1%, general internal medicine: 45.9%).

**Fig. 3:**
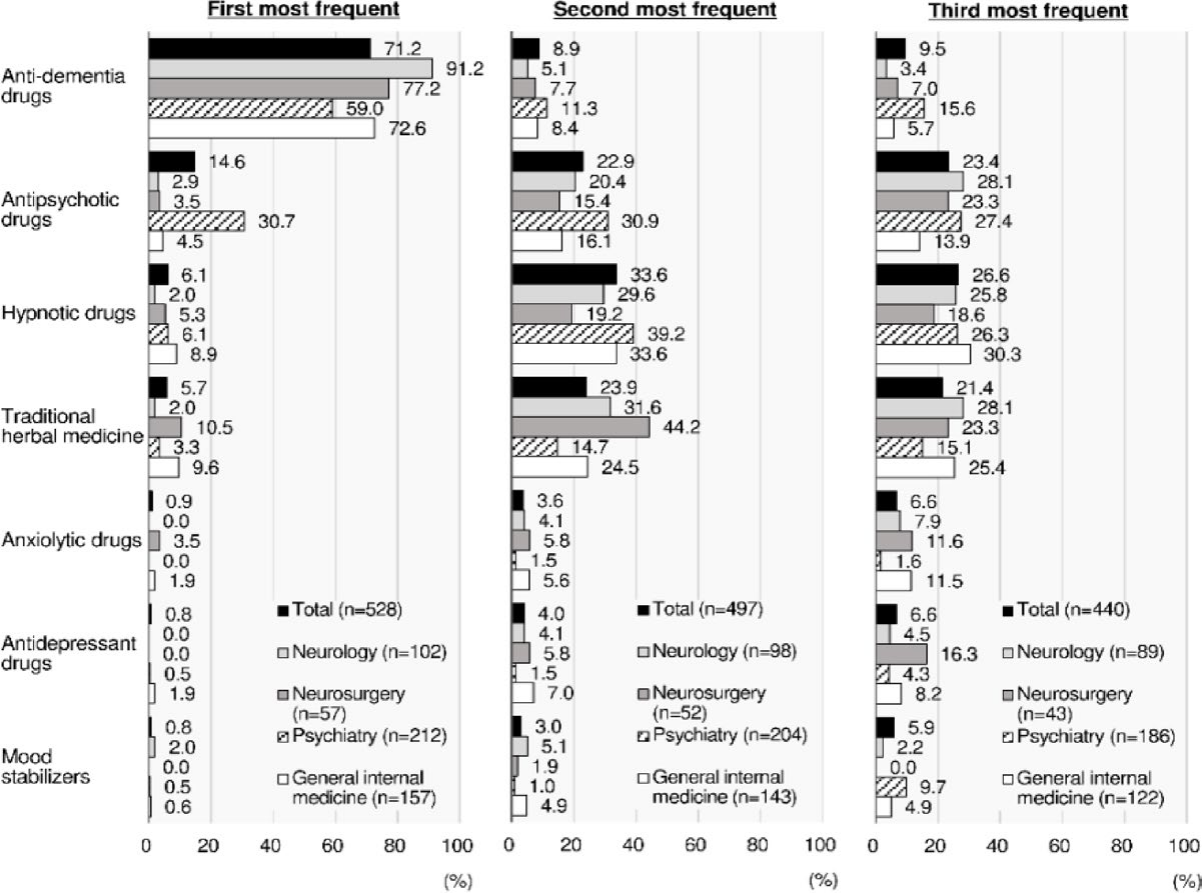
Top 3 medications newly prescribed for AD (*N* = 528)^a^. ^a^ Physicians who selected only “others” in the initial question (asking which medications they had newly prescribed for people with AD) were not shown this subsequent question, and are, therefore, not included in this figure (*n* = 1). The question specifically asked the physicians to select the first, second, and third most frequently prescribed medications among all newly prescribed medications for AD. AD, Alzheimer’s dementia.

Physicians indicated their first, second, and third most frequently prescribed medications among all newly prescribed medications for AD (*n* = 528), except one neurologist who selected “others” as medications for AD. Anti-dementia drugs were the most frequently prescribed medications overall (71.2%) and across specialties [Fig. 3]. The second most frequently prescribed medication varied by specialty: traditional herbal medicine in neurology (31.6%) and neurosurgery (44.2%); hypnotics in psychiatry (39.2%) and general internal medicine (33.6%). Antipsychotics ranked high in neurology, neurosurgery, and psychiatry as the third most frequently prescribed medication. Hypnotics and traditional herbal medicines ranked highly in neurology, neurosurgery, and general internal medicine.

#### Reasons for not prescribing antipsychotics for AD

Among the physicians who did not select antipsychotics as newly prescribed medications for AD treatment (*n* = 121), the most common reason was “not necessary or not urgent” overall (45.5%) and across specialties (neurology: 55.0%, neurosurgery: 32.0%, psychiatry: 33.3%, and general internal medicine: 49.3%). In psychiatry, “Safety concern other than side effect” and “off-label use” were more commonly reported (33.3% and 22.2%, respectively) relative to other specialties (neurology: 10.0% and 5.0%, respectively; neurosurgery: 4.0% and 4.0%, respectively; and general internal medicine: 7.5% and 6.0%, respectively). “Limited experience of usage” was relatively common in neurosurgery (28.0%) and general internal medicine (20.9%).

### Prescription behaviour for AAD

**Fig. 4:**
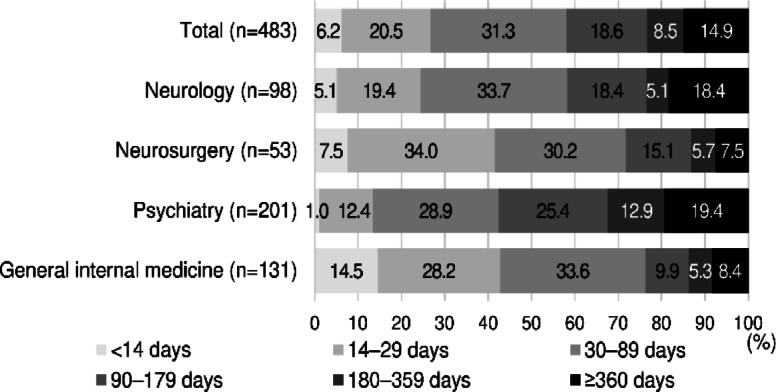
Duration of antipsychotic prescription among the physicians who prescribed antipsychotics for AAD (*N* = 483). Prescription duration from initiating to discontinuing antipsychotics for people with AAD: <14 days, 14–29 days, 30–89 days, 90–179 days, 180–359 days, and ≥ 360 days. AAD, Agitation in Alzheimer’s dementia.

Of the 492 physicians treating people with AAD, 69.5% answered that they prescribed or frequently prescribed antipsychotics for AAD. The percentage was higher in psychiatry (92.5%) than in other specialties (neurology: 62.6%, neurosurgery: 49.1%, and general internal medicine: 48.9%). Among the 483 physicians who answered that they prescribed antipsychotics to people with AAD, most physicians initiated the prescription because of “the degree of excessive motor behaviour or aggression” (88.2%) and “caregiver’s request” (52.4%). Meanwhile, most of them discontinued the treatment owing to “side effects” (76.0%) and “caregiver’s request” (53.8%) (by specialties, “side effects”: 76.5%, 79.2%, 79.1%, and 69.5%; “caregiver’s request”: 62.2%, 50.9%, 59.2%, and 40.5% in neurology, neurosurgery, psychiatry, and general internal medicine, respectively). The prescription duration was generally less than 90 days (58.0%) overall and across specialties, except for psychiatry [Fig. 4].

As key considerations for antipsychotic prescription, in the same subgroup of 483 physicians, “need for treatment or high urgency” was most common (58.0%), followed by “low risk of side effects” (57.1%). In psychiatry, the percentages citing “low risk of side effects” (68.2%) and “patient’s health status” (57.7%) were relatively high [Supplementary Fig. S1].

When prescribing antipsychotics, many physicians across specialties were concerned about somnolence and excessive sedation, and gait disturbance and unsteadiness [Supplementary Fig. S2]. Most psychiatrists were concerned about somnolence and excessive sedation (82.1%), gait disturbance and unsteadiness (82.1%), falls (79.1%), and dysphagia and dysarthria (76.1%); the last of which was notably higher than that in other specialties by approximately 38%. In contrast, in neurosurgery and general internal medicine, the percentage of physicians concerned about somnolence and excessive sedation (71.7% and 64.1%, respectively) was lower (> 10%) than that in other specialties. Generally, these specialties showed a smaller percentage of physicians reporting concerns for most side effects than other specialties.

## Discussion

This study was conducted to provide an overview of physicians’ perceptions of AAD and actual treatment practices in Japan, and revealed the variability in AAD recognition and treatment by specialty. The results suggest a gap in knowledge, experience, and practices related to diagnosis and pharmacological treatment for AAD, highlighting the need for standardization. Data on practice characteristics, including the number of people treated and practice setting (outpatient/inpatient), may offer valuable context for understanding the real-world clinical management of AAD in Japan.

Awareness and usage of BPSD measures were generally insufficient. Among the specialties included, the percentage of unawareness was the highest in general internal medicine. Although a small percentage, some of the internists were applying these measures, suggesting that they were striving to engage in BPSD treatment. In psychiatry, 17.5% reported being aware of and using these measures; approximately the remaining 80% did not employ the measures. Psychiatrists are generally considered to be familiar with AD and BPSD. This familiarity may have contributed to the underuse of BPSD measures, possibly resulting in suboptimal diagnosis and treatment. Based on these results, physician training and guidance are warranted to facilitate more systematic evaluation of BPSD.

Recognition of AAD was also low, particularly in general internal medicine. Relative to other specialties, fewer physicians in general internal medicine selected the correct agitation-related symptoms according to IPA’s definitions^13^(i.e., “disinhibition,” “irritability/lability,” “aberrant motor behaviour,” and “agitation/aggression (NPI)”), and 24.2% reported not being aware of AAD. More psychiatrists showed accurate understanding of AAD, with more frequent selection of “agitation/aggression (NPI)” and “irritability/lability” than other specialties (by > 15%). Nevertheless, as an overall trend, agitation has yet to be well-recognized in Japan. Given the substantial impact of AAD, timely and appropriate treatment is essential. However, insufficient awareness among physicians may delay diagnosis and treatment, and exacerbate caregiver burden. This variation may partly reflect the absence of an official Japanese translation of the IPA definition at the time of the survey. After completion of the survey, an official Japanese version was published^14^. Although this development is expected to improve disease recognition, translation should be followed by dissemination and education to enhance awareness and consistency in clinical practice. Our findings may help identify areas where targeted efforts are needed to address existing gaps.

The data revealed differences in prescription patterns for AD between specialties. Psychiatrists frequently selected antipsychotics, whereas other specialists preferred anti-dementia medications and tended to select hypnotics and traditional herbal medicines as the second and third most frequently prescribed medications. The choice of these medications may reflect attempts to address BPSD often observed and difficult to manage. Moreover, the findings may partly reflect differences in symptom severity or referral biases. Generally, psychiatrists are more likely to see patients with more severe or refractory behavioural symptoms, including those referred from other specialties after initial treatment turned out to be insufficient. Under these circumstances, antipsychotics may be one of the few therapeutic options available for psychiatrists to address these cases. Such clinical situations may have contributed to the observed differences in prescription patterns according to specialty. In specialties other than psychiatry, antipsychotics were less commonly prescribed for AAD and were typically used for shorter durations. The most common reason for not prescribing antipsychotics was “not necessary or not urgent,” with particularly high percentage in neurology and general internal medicine. However, many physicians reported that “agitation/aggression (NPI),” “aberrant motor behaviour,” “nighttime behaviours,” “irritability/lability,” and “delusions” were frequently observed and difficult to manage from both caregivers’ and their own perspectives. These data highlight the potential discrepancies between perceived needs and treatment decisions. Given that some people with mild cognitive impairment exhibit BPSD (13%–66%)^15,16^, physicians should consider the possibility of BPSD from the first consultation with people with AD. Assessment tools may serve as useful aids in this process.

When prescribing antipsychotics, many psychiatrists prioritized “low risk of side effects” and “patient’s health status” and paid attention to various side effects. The tendency toward higher awareness in psychiatry than in other specialties was notable for dysphagia and dysarthria. Psychiatrists may tend to prescribe antipsychotics carefully for people with AAD, considering the overall condition and treatment risks. Their careful attitudes may also be reflected in the relatively less frequent selection of anxiolytics for people with AD, possibly because of concerns about increased risk of cognitive dysfunction, delirium, fall, and fracture by anxiolytics, including benzodiazepines^17,18^.

Although many symptoms frequently observed and challenging for caregivers and physicians lie within the definition of agitation^13^, some physicians hesitated to use antipsychotics for AAD because of off-label use. Immediately before the present survey, the first AAD medication was approved in Japan. Further studies on potential treatment options may help promote better clinical recognition and education regarding AAD and its treatment. Such efforts are likely to become increasingly important considering the projected rise in the number of people with AD in Japan.

Caution should be exercised regarding the following limitations. First, survey panel registrants may be more aware of these topics than non-registrants, limiting the representativeness of the selected samples. Second, as a self-administered survey, the results are subject to response biases such as recall and social desirability biases, which may lead to over- and under-reporting of desirable and undesirable behaviours, respectively. Furthermore, the response rate was 15.0% (533/3,560). Therefore, the possibility of non-response bias cannot be excluded, and potential differences between respondents and non-respondents may limit the generalizability of the findings.

## Conclusions

This is the first survey to describe the perceptions and treatment practices for AAD in current Japanese clinical settings. The responses revealed variations by specialty, particularly high frequency of antipsychotic prescriptions with higher awareness of the risk of side effects among psychiatrists, and limited recognition of AAD, especially in general internal medicine. These findings highlight knowledge gaps that must be addressed to improve clinical practice. In the context of limited understanding of the actual status of AAD in Japan, these data will serve as a foundation for future research and awareness initiatives to enhance understanding and optimal management of AAD across medical specialties.

## Electronic Supplementary Material

Below is the link to the electronic supplementary material.

Supplementary Material 1

## Data Availability

The datasets generated and/or analysed during the current study are not publicly available due to contractual restrictions with the survey panel provider but are available from the corresponding author on reasonable request.

## References

[1] Ninomiya, T. Ninchisho oyobi keido ninchi shogai no yubyoritsu chosa narabi ni shorai suikei ni kansuru kenkyu hokokusho (in Japanese). (2023). https://www.eph.med.kyushu-u.ac.jp/jpsc/uploads/resmaterials/0000000111.pdf?1715072186

[2] Alzheimer’s Association. 2025 Alzheimer’s Disease Facts and Figures. Alzheimers Dement. 21, e70235; 10.1002/alz.70235 (2025).

[3] International Psychogeriatric Association. The IPA complete guides to behavioral and psychological symptoms of dementia. (2010). https://www.ipa-online.org/resources/publications/guides-to-bpsd

[4] Pinyopornpanish, K. et al. Impact of behavioral and psychological symptoms of Alzheimer’s disease on caregiver outcomes. *Sci. Rep.* **12**, 14138 (2022). 35986203 10.1038/s41598-022-18470-8 PMC9391353

[5] Selbaek, G., Engedal, K. & Bergh, S. The prevalence and course of neuropsychiatric symptoms in nursing home patients with dementia: a systematic review. *J. Am. Med. Dir. Assoc.* **14**, 161–169 (2013). 23168112 10.1016/j.jamda.2012.09.027

[6] Halpern, R. et al. Using electronic health records to estimate the prevalence of agitation in Alzheimer disease/dementia. *Int. J. Geriatr. Psychiatry*. **34**, 420–431 (2019). 30430642 10.1002/gps.5030 PMC7379654

[7] Sano, M. et al. Agitation in cognitive disorders: Progress in the International Psychogeriatric Association consensus clinical and research definition. *Int. Psychogeriatr.* **36**, 238–250 (2024). 36880250 10.1017/S1041610222001041 PMC10684256

[8] Jones, E. et al. Agitation in Dementia: Real-World Impact and Burden on Patients and the Healthcare System. *J. Alzheimers Dis.* **83**, 89–101 (2021). 34250934 10.3233/JAD-210105 PMC8461728

[9] Schein, J. et al. The Impact of Agitation in Dementia on Caregivers: A Real-World Survey. *J. Alzheimers Dis.* **88**, 663–677 (2022). 35694920 10.3233/JAD-215670 PMC9398061

[10] Research Group for the Development of Clinical Guidelines on New Anti-Aβ Antibody Drugs for Alzheimer’s Disease and Psychotropic Medications for BPSD. (2024 Health and Labour Sciences Research Grant). Guideline for the use of psychotropic medications to manage BPSD for primary care physicians and dementia support doctors. 3rd ed. (in Japanese). (2025). https://dementia-japan.org/wp-content/uploads/2025/06/guideline.pdf

[11] Ministry of Health. Labour and Welfare. Survey on Appropriate Use of Psychotropic Drugs by Primary Care Physicians for Dementia Patients (in Japanese). (2021). https://mhlw-grants.niph.go.jp/project/25227

[12] Otsuka Pharmaceutical Co., Ltd. Otsuka Obtains Additional Indication for Rexulti® in Japan as an Adjunctive Treatment for Agitation Associated with Dementia due to Alzheimer’s Disease. https://www.otsuka.co.jp/en/company/newsreleases/2024/20240924_2.html (2024).

[13] De Mauleon, A. et al. Agitation in Alzheimer’s disease: Novel outcome measures reflecting the International Psychogeriatric Association (IPA) agitation criteria. *Alzheimers Dement.* **17**, 1687–1697 (2021). 34132461 10.1002/alz.12335 PMC9292260

[14] Ikeda, M. et al. Agitation in Japan: an overview and the Japanese definition. (in Japanese). *Dementia Japan.* in the press (2026).

[15] Kohler, C. A. et al. Neuropsychiatric Disturbances in Mild Cognitive Impairment (MCI): A Systematic Review of Population-Based Studies. *Curr. Alzheimer Res.* **13**, 1066–1082 (2016). 27137220 10.2174/1567205013666160502123129

[16] Yamaguchi, H. et al. Trends of BPSD, evaluated with NPI, in outpatients of the Medical Center for Dementia. (in Japanese). *Tokyo J. Dement. Care Res.* **1**, 3–10 (2017).

[17] Airagnes, G., Pelissolo, A., Lavallee, M., Flament, M. & Limosin, F. Benzodiazepine Misuse in the Elderly: Risk Factors, Consequences, and Management. *Curr. Psychiatry Rep.* **18**, 89 (2016). 27549604 10.1007/s11920-016-0727-9

[18] Takahashi, K. et al. Changes in the Dose of Benzodiazepines and Falls in Elderly Inpatients in an Acute-care Hospital. *Jpn J. Pharmacoepidemiol*. **16**, 11–20 (2011).

