# Supplementary material for: Physicians’ perceptions and treatment practices for agitation associated with Alzheimer’s dementia vary by specialty in Japan
Source: Sci Rep. 2026 May 7;16:21081. doi: 10.1038/s41598-026-51118-5 (PMC13342074; doi:10.1038/s41598-026-51118-5)
Supplement: Supplementary file 1 — Supplementary Material 1 [file 41598_2026_51118_MOESM1_ESM.docx]

# **Supplementary Figure S1.** **Key considerations in prescribing antipsychotic drugs among physicians who prescribed antipsychotics to people with AAD (N=483)**


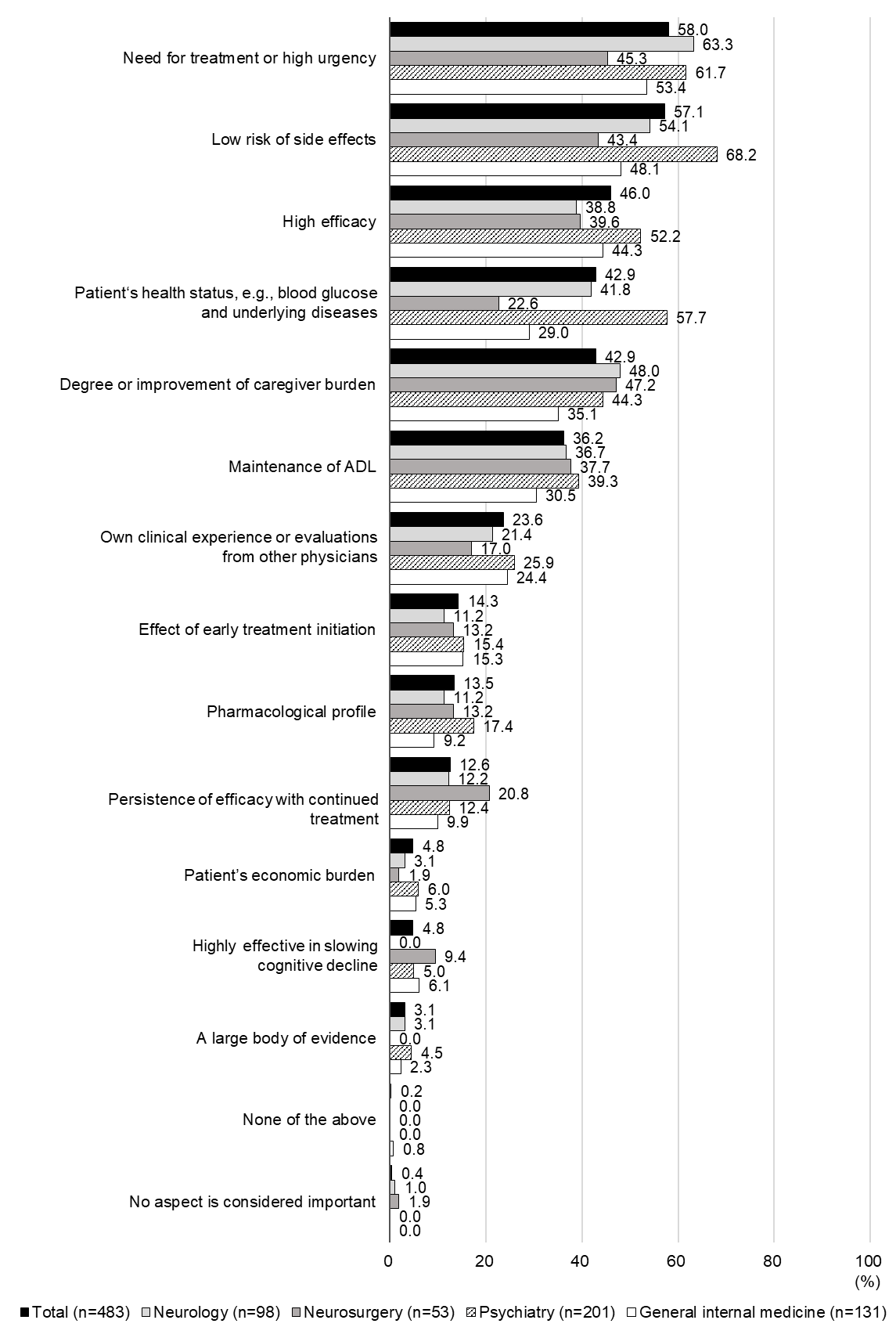


The question asked physicians to select all the key considerations upon prescribing antipsychotics to people with AAD from the choices presented in the figure.

AAD, Agitation in Alzheimer's dementia; ADL, Activities of daily living

# **Supplementary Figure S2. Side effects concerned upon prescribing antipsychotic drugs for people with AAD (N=483)**


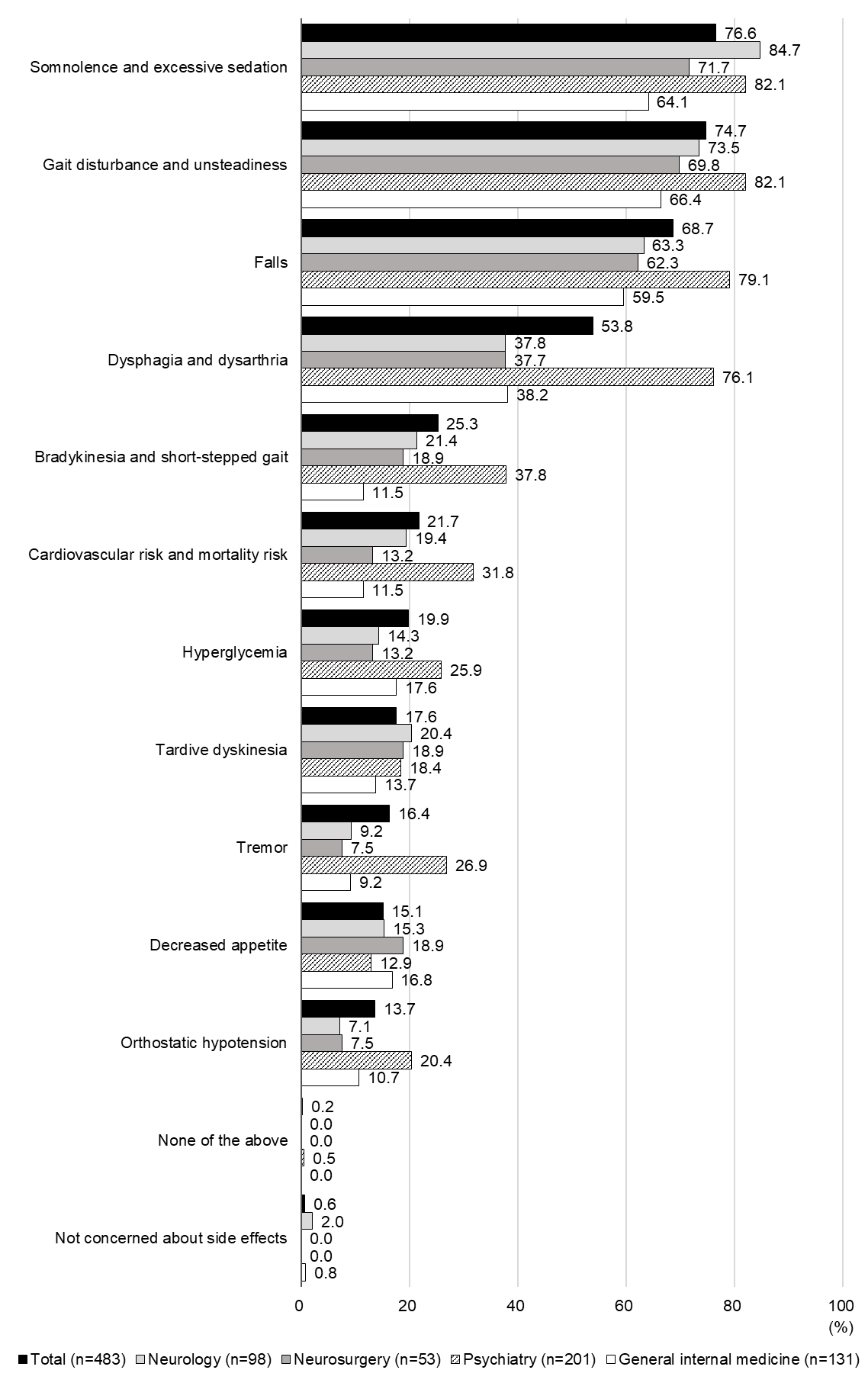


The question asked physicians to select all the side effects concerned upon prescribing antipsychotics to people with AAD from the choices presented in the figure.

AAD, Agitation in Alzheimer's dementia
